# Characterization of the Alkali and Hydrolysis Resistance of Polymer-Impregnated, Alkali-Resistant Glass Filaments

**DOI:** 10.3390/ma17174343

**Published:** 2024-09-02

**Authors:** Florian Kempis, Jeanette Orlowsky

**Affiliations:** Department of Building Materials, Faculty of Architecture and Civil Engineering, TU Dortmund University, August-Schmidt-Str. 8, 44227 Dortmund, Germany; jeanette.orlowsky@tu-dortmund.de

**Keywords:** alkali resistance, AR glass, durability, hydrolysis resistance, polymers, styrene-butadiene rubber, SBR, thermoplastic, textile concrete

## Abstract

The aim of this series of tests was to characterize the alkali and water resistance of alkali-resistant (durability) glass filaments, which were optimized with two non-vulcanized formulations based on co-polymerizing styrene-butadiene rubbers (CemFil-SBR1 and CemFil-SBR2). Furthermore, it was assessed which of the two polymer-impregnated multifilament yarns is the better alternative for use in cementitious binders. For this purpose, the impregnated multifilament yarns were chemically conditioned for up to twelve months at temperatures of 23 and 50 °C in 2.5 percent sodium hydroxide solution and 2.5 percent potassium hydroxide solution as well as in 3 percent salt and distilled water. The samples were then subjected to material science tests. The liquid absorption capacities and the changes in the mass of the composite materials were determined at different times during conditioning. The load-bearing capacity of the samples was also tested using uniaxial fiber strand tensile tests. The durability of the polymer-impregnated multifilament yarns was described in detail in conjunction with scanning electron microscopy images and nominal cross-section determinations. The test liquids caused a reduction in strength during the storage period, which was accelerated by increased temperatures. The reduction in strength is mainly due to glass corrosion of the filaments. Glass corrosion is delayed due to the good impregnation quality, which fundamentally improves the durability of the yarns. The results of the durability tests show that the polymer-impregnated multifilament yarns CemFil-SBR2 are probably more suitable for use in cementitious binders, as they have better alkali and hydrolysis resistance.

## 1. Introduction

The outstanding mechanical and physical properties of concrete make it the most widely used building material in the world. In combination with steel (reinforced concrete), concrete is able to withstand high compressive and tensile stresses.

This success of concrete as a building material is accompanied by considerable disadvantages. The production of cement clinker causes the emission of climate-relevant gases. Ammonia (NH_3_), mercury (Hg), sulfur dioxide (SO_2_), nitrogen oxides (NO_x_), and others are continuously emitted into our atmosphere during the manufacturing process. Added to this is the most common greenhouse gas: carbon dioxide (CO_2_). The production of one ton of cement releases around 600 kg of CO_2_ into the atmosphere [1].

Worldwide, between 4.05 and 4.6 billion tons of cement have been produced and consumed annually over the last ten years, releasing around 2.8 billion tons of harmful CO_2_ into the atmosphere [2,3].

The production of one ton of crude steel in connection with concrete production generates around 1.7 tons of CO_2_ and additionally pollutes the environment through its emission [4].

Furthermore, aggregates are used to produce concrete, which is extracted from natural (and therefore finite) deposits. Added to this is the consumption of considerable quantities of (drinking) water.

At the end of the production chain, the concrete components prefabricated by the industry have to be transported to the construction sites for further processing [5].

These factors reveal the direct link between concrete production and processing and climate change, accelerated by humans.

The steel embedded in the concrete can be replaced by multifilament yarns (“rovings”) or technical textiles (scrims or fabrics) made of carbon fibers or alkali-resistant glasses (AR glasses), for example. This composite material is known as textile concrete. It has several advantages with comparable mechanical properties to classic concrete.

Steel corrosion does not exist for textile concretes. This can result in a longer service life for the concrete components. 

Textile concrete allows the production of thinner and therefore lighter components; this saves considerable resources (cement content, aggregate) and costs (transportation costs, etc.).

The multifilament yarns or the scrims or fabrics made from them are often impregnated with various impregnation systems (e.g., modified epoxy resins or synthetic polymer dispersions) to increase the bond strength between reinforcements and binders.

Reinforced concrete components can be subsequently further reinforced using textile-reinforced concrete. It is possible to support areas of existing concrete components (e.g., ceiling beams) that are stressed by tensile stresses with a multi-layer textile concrete system. Furthermore, textile-reinforced concretes allow subsequent sealing options for reinforced concrete components where unplanned water-bearing cracks have occurred. Such water-bearing crack widths can be reduced to a permissible (non-water-bearing) width for the service condition using a textile-reinforced concrete system, thus ensuring permanent impermeability. A prominent example is the repair of the reinforced concrete roof surfaces of the Mariendom in Velbert-Neviges [6,7,8].

The acquisition costs of sized, alkali-resistant glass fibers are on average one-fifth cheaper per kilogram than the more expensive carbon fibers. Furthermore, glass fibers have a low CO_2_ footprint, which makes their use more economical and sustainable. 

The majority of AR glasses used for steel substitution in concretes are based on a ternary SiO_2_–ZrO_2_–Na_2_O system [9].

However, in addition to the advantages described, AR glass also has a disadvantage relevant to construction technology.

Using the SIC test, Litherland established that the reduction in the tensile strength of glass fibers is due to a corrosive attack by the alkaline environment prevailing in concrete [10,11]. In this respect, AR glass does not have complete resistance to alkaline (concrete pore) solutions. To put it more precisely, AR glasses are merely more resistant in the long term than other types of glass (e.g., E-glasses). This higher resistance of commercial AR glasses is due to a zirconium oxide content (ZrO_2_) of between 16 and 20% by weight [12].

In alkaline solutions, an AR glass with a higher ZrO_2_ content loses less bound sodium and silicon from its amorphous structure (glass network) than an AR glass with a lower ZrO_2_ content. 

Simhan assumes a diffusion-controlled (chemical) process [13]. Purnell also assumes a chemical attack on the glass network [14]. Different temperatures, pH values of pore solutions, and stress concentrations cause a parameter-dependent growth of defects on the reinforcement surface (cf. “Static Fatigue Model” [15]).

Orlowsky’s work shows that the corrosive process is mainly due to an initially solution-controlled splitting of the glass network, which is successively replaced by a diffusion-controlled process [16].

On the other hand, Butler concludes that the strength losses are mainly caused by changes in the contact zone between the AR glass and the surrounding cement matrix [17,18]. 

During the hydration of concrete, CSH phases press into the glass structure, resulting in mechanical damage to individual filaments. Stucke, Majumdar, and Bentur also described a change in the microstructure matrix around the glass fibers, which they used to explain the damage or loss of strength [19,20]. However, the authors mentioned did not fundamentally rule out the occurrence of defects. Nonetheless, they did not ascribe as much importance to imperfections as Purnell and Orlowsky.

Although AR glasses are more resistant to alkaline solutions, commercially available glass fiber-reinforced plastics (GRP) based on E or ECR glasses are used in the construction industry for the most part. GFRPs are often impregnated with vinyl ester resins [21,22].

E-glasses react with concrete pores (water) and form hydroxides, among other things [23]. The hydroxides reduce the pH value of the concrete pore solution, which in turn results in the decomposition (from a pH value of approximately 9) of Si–O–Si bonds of the glass network [24]. This process ends in a significant loss of strength.

In the literature, very different test parameters (primarily temperature and time) and various alkaline test liquids have been used to investigate the durability of glass. In most cases, the glass types (E, ECR, AR glass) were exposed to different percentages of KOH, NaOH, Ca(OH)_2_ solutions or filtered cement solutions in order to investigate polymorphic corrosion phenomena on the individual filaments. The multifilament yarns were stored in the different test liquids for periods of one to 200 days at temperatures between 20 and 100 °C [25,26,27].

The H^+^ cations and OH^−^ anions present in water and alkaline solutions interact with the amorphous glass network on a molecular level. The processes of water attack on glass are mainly based on ion exchange processes, whereby alkali ions are dissolved out of the glass structure. This leads to the accumulation of OH^−^ anions in the corrosion area, resulting in a (silica) gel layer on the glass surface due to the condensation of Si–OH groups. This gel layer describes a corrosion rim about 50–200 nm thick, which is most likely formed by the hydrolysis of the glass network. The corrosion rim contains molecular water and is characterized by good ion mobility, which is why it is also referred to as a diffusion zone [28,29,30,31]. In contrast to an alkaline attack by alkaline solutions (“lyes”) on the glass structure, this diffusion-controlled reaction takes place extremely slowly. The oxygen bridge bonds of the glass network are split by OH^−^ anions in the alkaline environment so that the glass structure is dissolved. The reaction kinetics of the alkaline attack increases with increasing pH value and can be described with the following reaction equation according to [32]:

≡ Si–O–Si ≡ +OH^−^ → ≡ Si–O^−^ + ≡ Si–OH ≡ Si–O^−^ + H_2_O → ≡ Si–OH + OH^−^

To summarize, the various physico-chemical corrosive processes lead to a loss of strength of E-glass, AR-glass, or AR-glass filaments and textiles.

The corrosion phenomena and intensities are obviously diverse and are controlled by several parameters such as temperature and time, the concrete pore solution, and the material composition of the glass. In particular, the quality of the impregnation system has an enormous influence on the rate of strength loss [33].

The aim of this series of tests is to assess the alkali and hydrolysis resistance of polymer-impregnated AR glass multifilament yarns that have been chemically conditioned over a defined period of time. 

The multifilament yarns were impregnated with two aqueous polymer dispersions based on co-polymerizing, non-vulcanized styrene-butadiene rubbers (hereinafter CemFil-SBR1 and CemFil-SBR2). The polymeric impregnation system SBR2 showed the best alkali and hydrolysis resistance in preliminary tests, which makes it more suitable for use in cementitious binders [33].

However, the viscosity and surface tension of the two aqueous polymer dispersions, among other factors, are decisive for the impregnation quality of the multifilament yarns. The viscosity and surface tension ultimately determine the degree of penetration of the individual filaments into the core of the multifilament yarns. They also have an effect on the wettability and adhesion of the aqueous polymer dispersions on the glass surface. According to this fact, it is conceivable that the SBR1 impregnation system (despite a lower alkali and hydrolysis resistance) wets individual filaments of the textiles more completely due to a more suitable surface tension and lower viscosity. This, in turn, could increase the performance of the polymer system in cementitious binders, as significantly more filaments are involved in load transfer.

## 2. Materials and Methods

### 2.1. Sample Material, Chemical Conditioning, and Sample Preparation

The multifilament yarns used in this test series are AR glass direct rovings of the type Cem-Fil© 5325 from Owens Corning in Toledo (OH, USA) with an average filament diameter of 14 µm, a specific weight of 2.68 g/cm^3^, and a fineness of 640 tex. The organic impregnations SBR1 and SBR2 were specially developed by the CHT Germany Group in Tübingen (Baden-Württemberg, Germany) for the impregnation of AR glass to increase resistance in alkaline media. The aforementioned multifilament yarns were impregnated there with the two different impregnations. The approximately one-meter-long impregnated multifilament yarns were cut into 50 mm and 560 mm long segments with a scalpel so that they would fit into the different storage media.

The 50 mm long samples were stored airtight for up to one year in polypropylene containers at 23 °C and 50 °C, which were filled with various alkaline test liquids and distilled water (Table 1). The determination of the nominal cross-section (Section 2.2), the testing of the liquid absorption capacity (Section 2.3), the thermogravimetric analyses (Section 2.4), and the microscopic examinations (Section 2.5) were carried out on these samples.

The 560 mm long segments were placed in glass storage media (threaded jars) specially made for the conditioning, with the help of which the area of the defined free test length or clear length *l*_0_ = 340 mm, which is only relevant for the tensile strength tests (Section 2.5), is affected by the corresponding test liquids.

The pH value of the test liquids was checked regularly using the pH meter CG 822 from Schott Geräte GmbH, Mainz (Rheinland-Pfalz, Germany).

The samples were mostly removed from storage after 7, 14, and 28 days (short-term conditioning) and after 6 and 12 months (long-term conditioning). The exception is chemical conditioning for the purpose of investigating liquid absorption capacity (see Section 2.3). Following storage, all samples were stored in a desiccator at room temperature for 24 h until the respective material science tests (preconditioning). The non-stored reference samples were stored airtight in glass containers, which were also protected from UV radiation, until preconditioning.

Note: Attempts were made to chemically condition and subsequently characterize non-impregnated multifilament yarns. However, the non-impregnated multifilament yarns in the various test liquids disintegrated into filament groups of different sizes after a very short time; in principle, this is attributed to the degradation of the sizing. The non-impregnated yarns could be chemically conditioned using a different test set-up. Scheffler “loosely” wrapped the yarn sections embedded in alkaline solutions with alkali-resistant glass threads [12]. This prevented individual filaments or filament groups from detaching from the yarn sections due to rinsing processes. The inclusion of such a further (different) test set-up in the methodological approach used in this work was dispensed with, as the resulting data would not have been unconditionally comparable.

The tensile strength of non-impregnated multifilament yarns, on the other hand, was tested (see Section 3.5).

### 2.2. Nominal Cross-Sections

To achieve an effective impregnation quality, an impregnation system should (among other things and as already mentioned) have low viscosity for optimum penetration of the individual filaments or textiles into the multifilament core as well as good wettability and adhesion to the material surface. A high air void content and/or different impregnation thicknesses (e.g., the formation of agglomerations) can have a detrimental effect on the performance of polymer impregnation systems. In other words, the more homogeneous and thicker the nominal cross-section of the impregnated multifilament yarns, the more effective the retardation of glass corrosion in alkaline media can be. 

Accordingly, the average nominal cross-section of the impregnated multifilament yarns CemFil-SBR1 and CemFil-SBR2 was determined based on the International Organization for Standardization [34]. The experimentally determined nominal cross-sections can also be used to calculate the densities of the two impregnation systems, SBR1 and SBR2. These characteristic values, in conjunction with the other material science results, allow the impregnation quality to be assessed and the protective effect of the polymer layer against the alkaline media to be estimated.

Twenty 50 mm long fiber strands of each of the impregnated multifilament yarns were cut to size to determine the nominal cross-sections. The sample volumes were then determined according to the principle of water displacement. The volume displacement of the fiber strands was measured at room temperature using a titration burette.

The fiber cross-section *A*_F_ and the nominal cross-section *A*_N_ in [mm^2^] were determined according to [35] using the following formula:

$$A_{F,N}=\frac{\left(V_{s}-V_{0}\right)\times 100}{l_{0}}$$

where:

*V*_s_—volume of water and sample in [mL];
*V*_0_—volume of water without sample in [mL];
*l*_0_—length of the sample in [mm].

The impregnation cross-section results from the difference between the nominal cross-section and the fiber cross-section including the air void content *A*_I_.

$$A_{I}=A_{N}-A_{F}$$

The fiber, nominal, and impregnation cross-sections were then multiplied by the defined length of 1000 mm to calculate the volumes *V*_N,F,I_.

$$V_{N,F,I}=\left[\left(A_{N,F,I}\times 0.01\right)\times 100\right]$$

Using the volume *V*_F_ and the fiber density of 2.68 g/cm^3^ known from the product data sheet, the masses of the non-impregnated fiber *m*_F_ could be calculated as follows:

$$m_{F}=\left[\left(A_{F}\times 0.01\right)\times 100\right]\times \rho_{F}$$

The masses of the impregnated rovings *m*_N_ were determined using the Plus analytical balance (model PA214C) from Ohaus Europe GmbH in Nänikon (Zurich), Switzerland. The difference between the weighed impregnated rovings *m*_N_ and the calculated mass of the non-impregnated fiber *m*_F_ gives the mass of the pure impregnation *m*_I_ (without air voids).

$${m_{I}=m}_{N}-m_{F}$$

The density of the impregnations *ρ*_I_ can then be calculated as follows.

$$\rho_{I}=\frac{m_{I}}{V_{N}}$$

### 2.3. Liquid Absorption Capacity

The liquid absorption of the co-polymerizing styrene-butadiene rubbers SBR1 and SBR2 is initially a physical aging process, which triggers a softening effect and a surface-enlarging swelling of the polymer structure [33].

In the case of the impregnated multifilament yarns CemFil-SBR1 and CemFil-SBR2, it is also conceivable that liquid can accumulate between the interfaces of the filaments, the sizing, and the polymer structures due to activated diffusion processes and submicroscopic capillary processes. This in turn would lead to a weakening in the adhesive bond between the different material phases, which would promote a loss of strength. 

The aim of the study was to test whether the impregnated multifilament yarns CemFil-SBR1 and CemFil-SBR2 have a material-dependent (varying) liquid absorption capacity. In addition, it should be clarified when the actual saturation limit of the polymer structures for the respective test liquids is reached.

**Figure 1:**
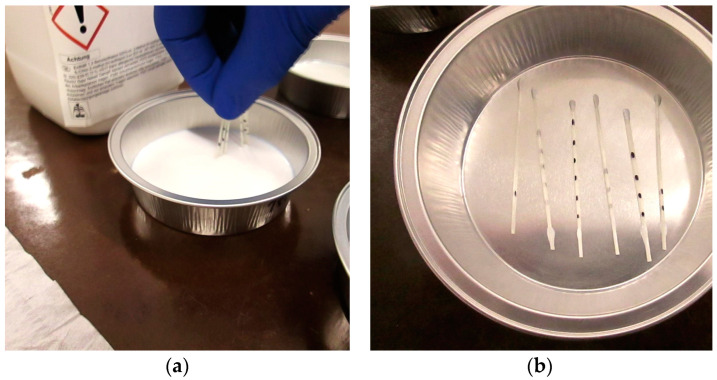
(**a**) Manual impregnation of several polymer-impregnated multifilament yarns cut to size; (**b**) general view of yarns in an aluminum tray before polymer synthesis in the climate chamber.

The ends of the 50 mm long, segmented yarns were additionally impregnated by hand with the respective aqueous polymer dispersions (Figure 1a). This measure was taken to ensure that the result of the liquid absorption capacity was not falsified by capillary suction processes on the two cut surfaces of the multifilament yarns. The aqueous polymer dispersions at the yarn ends of the samples were synthesized in a covered aluminum tray for a period of 24 h at a temperature of 23 °C and 50% relative humidity in a climate chamber (Figure 1b). 

The impregnated multifilament yarns CemFil-SBR1 and CemFil-SBR2 were dried to constant mass at 50 °C before storage. This process took no longer than 24 h. The test specimens were then placed in PP wide-neck bottles, which were then filled with the various test liquids and sealed. The impregnated multifilament yarns in the containers were then stored in two drying ovens at 23 °C and 50 °C. 

The amount of liquid absorbed by the polymer structure was calculated according to the work of [33].

The changes in mass are given as the arithmetic mean of three individual weighings, which were determined after 24, 48, 72, and 96 h as well as 7 and 28 days and after 3, 4, 5, and 6 months.

### 2.4. Thermogravimetric Analysis (TGA)

In Kempis’ work, only the changes in the mass of the free polymer films SBR1 and SBR2 (single-phase systems) after chemical conditioning were investigated [33]. However, the extraction of water-soluble components from the polymer films cannot in principle be transferred to the degradation of the polymer systems applied to the glass filaments. The CemFil-SBR1 and CemFil-SBR2 samples tested in this series of tests are multiphase systems consisting of the bond between the glass substrate CemFil 5325 and a coating applied to it and the respective impregnation systems SBR1 and SBR2. Due to the different manufacturing processes, the impregnated multifilament yarns have a smaller cavity volume than the free polymer films. This means that changes in mass due to external causes of aging of the different sample bodies cannot be compared with each other without hesitation. Furthermore, the sodium hydroxide and potassium hydroxide solution cause the dissolution of the glass network, which in turn is accompanied by the formation of corrosion products (cf. Section 3.4). These corrosion products or the dissolution of the glass network also have a detrimental effect on the balance of the mass change of the embedded yarns. The different sample geometries also lead to a disparate permeation behavior of liquids through the cross-section of the polymer structures or the entire material; however, this again makes it difficult to compare the changes in mass under the defined chemical conditioning scenario.

The thermogravimetric measurements are mainly used to record the changes in mass of the two chemically conditioned multiphase systems CemFil-SBR1 and CemFil-SBR2. The mass changes caused by the various external (and internal) causes of aging serve as a basic prerequisite for assessing the physico-chemical (polymeric) protective layer of the glass filaments. This protective layer reduces the mechanical stress on the filaments caused by the crystallites of the binder and slows down the permeation of alkaline pore solutions, which increases the serviceability of the glass filaments. Accordingly, the thermogravimetric measurement data are used to evaluate the technical service life of the material, as the efficiency of the polymer protective layer inevitably decreases with increasing mass loss.

Depending on a changing heating rate, the mass changes of up to five individual samples per conditioning scenario were analyzed using the TGA/DSC3+ from Mettler Toledo in Greifensee (Zurich), Switzerland. At the beginning of the method, the samples were heated to 105 °C at a heating rate of 20 K/min to evaporate free water in the cavity volume of the polymer structure. The temperature of 105 °C was maintained for 10 min. According to a representative number of preliminary tests with the chemically conditioned polymers, no change in mass could be detected after about 5 min. 

In the next step, a temperature of 250 °C was applied at a heating rate of 20 K/min. This temperature was also maintained for 10 min in order to remove volatile components (solvents, monomers, test liquids).

In the final step, the samples were heated at a heating rate of 10 K/min to a temperature of 650 °C, where the polymer structures and the coating applied to the glass filaments began to melt and subsequently decompose almost completely. The samples were annealed at the final temperature of 650 °C for 10 min. Higher temperatures did not lead to deviating mass changes. 

The changes in the mass of the conditioned samples were evaluated in accordance with DIN 51006 (one-step mass changes) using the STARe Excellence software (version 16.30) from Mettler Toledo, Zurich, Switzerland [36].

### 2.5. Microscopy

The scanning electron microscopic examination of the impregnated multifilaments was used to optically describe the (surface) degradation of the impregnation systems caused by the test liquids. 

The scanning electron microscope images were generated using the FIB-REM Crossbeam XB 550L from Zeiss in Oberkochen (Baden-Württemberg), Germany. The sample surface of the impregnated multifilament yarns was dusted with an approximately 5 nm thick layer of graphite to prevent charging (deposition) using a DC sputtering system. The samples were then viewed in the low voltage range of 5 kV at a working distance (WD) of between 7.2 mm, 8.2 mm, 11.1 mm, 11.2 mm, 12.1 mm, and 12.2 mm. Secondary electrons were used to generate images, which were detected with an SE2 detector (Everhart-Thornley detector).

### 2.6. Fiber Strand Tensile Tests

The resistance of AR glass to alkaline (or acidic) media cannot be clearly tested or measured. 

Accordingly, it is only possible to compare different (impregnated) AR glasses with each other and characterize them accordingly. The reason for this limitation is that changes in AR glass structures depend on many factors (see Section 1, Introduction).

The primary aim of the fiber strand tensile tests was to assess the degree of protection of the two impregnation systems SBR1 and SBR2 and the associated durability of the chemically conditioned multifilament yarns CemFil-SBR1 and CemFil-SBR2. The protective effect describes the delay of the corrosive processes within the AR glass structure caused by the polymer structures, which are triggered by the sodium hydroxide and potassium hydroxide solution. The effects due to chemical changes in the polymer structures caused by the influence of these alkaline test liquids on the tensile strength of the impregnated filaments cannot be clearly characterized due to the superimposed corrosion processes. For this reason, the structure-changing effects on the impregnated yarns and their tensile strength, which are triggered by salt and distilled water, were also recorded.

In contrast to tensile strength tests on composite samples (meaning: polymer-impregnated textiles in cementitious binders), mechanical damage to individual filaments or to the impregnation systems caused by crystallizing mineral phases of a cement matrix can be ruled out in fiber strand tensile tests. Furthermore, the effect of different thermal expansion coefficients due to a hydrating binder matrix on the material can be neglected. According to this fact, the performance and durability of the impregnated multifilament yarns can be meaningfully assessed. 

The quality of the bond between filaments and impregnation systems can also be assessed on the basis of the results of the fiber strand tensile tests in conjunction with the thermogravimetric analyses and the scanning electron micrographs.

**Figure 2:**
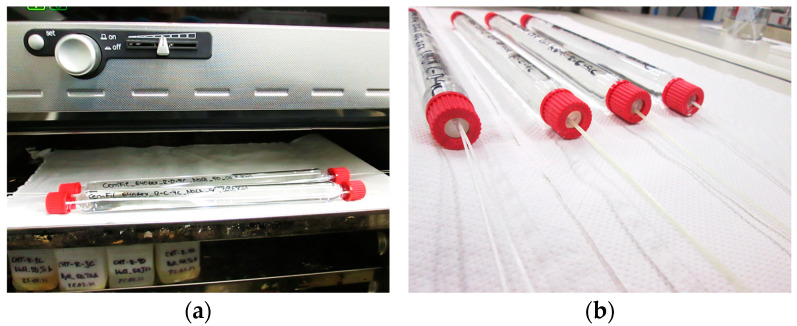
(**a**) Storage of two threaded jars with multifilament yarns bound in them in the 50 °C climate chamber; (**b**) removal of threaded jars with multifilament yarns embedded in them after chemical conditioning.

**Figure 3:**
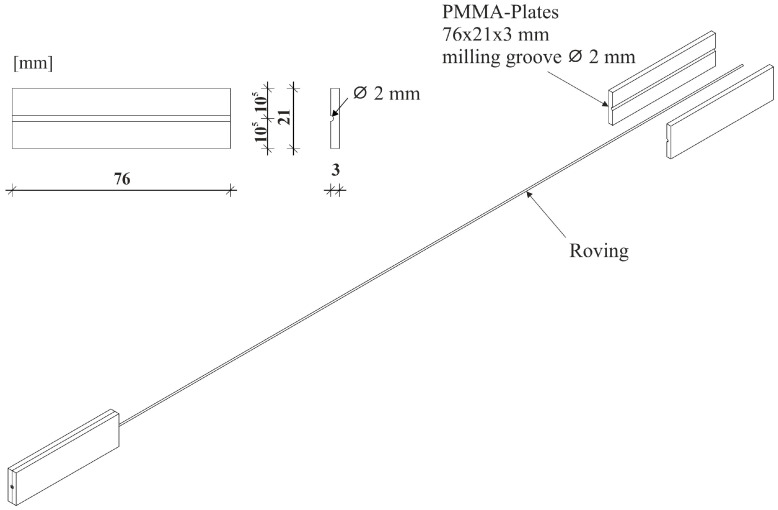
Technical drawing of the PMMA-Plates.

Following chemical conditioning (see Figure 2), the two ends of the extracted multifilament yarns were inserted into 1.5 mm milled grooves in two test plates made of polymethyl methacrylate (PMMA). The dimensions of the test plates are 76 mm × 21 mm × 3 mm. The filament ends inserted in the grooves were bonded with the universal instant adhesive Ropid 150 (Toolcraft) from Conrad Electronic SE in Hirschau (Bavaria), Germany. Subsequently, two further test plates of the same shape were glued flush onto the test plates fixed with the filament ends (Figure 3).

**Figure 4:**
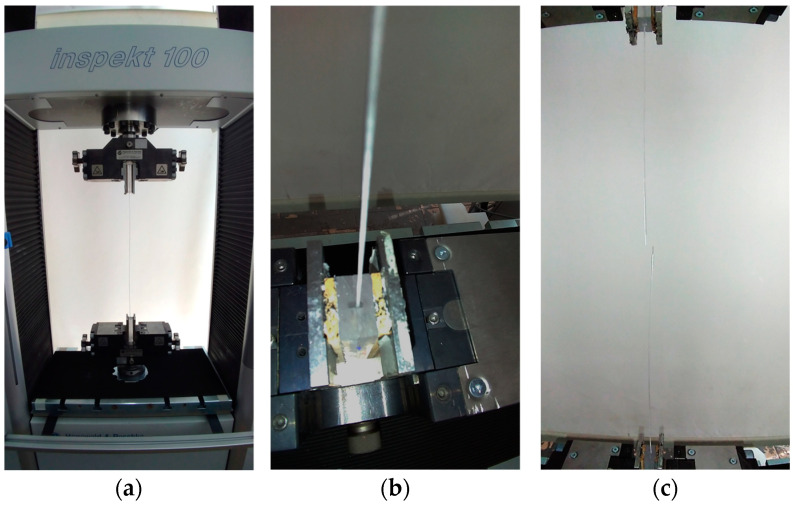
(**a**) Fiber tensile test using the Inspekt 100 universal testing machine from Hegewald and Peschke; (**b**) the end of the multifilament yarn is embedded in the adhesive bed and has no direct contact with the two PMMA-Plates; (**c**) due to the sample preparation, the majority of the multifilament yarns failed in the free path length segment.

To determine the tensile strength of the impregnated multifilament yarns, up to ten individual samples were tested for each conditioning scenario using the Inspekt 100 universal testing machine from Hegewald und Peschke Mess- und Prüftechnik GmbH in Nossen (Saxony), Germany (Figure 4a). The force applied during the test was related to the fiber cross-sectional areas of the multifilament yarns specified by the manufacturer.

The stress peaks on the transverse pressure-sensitive AR glass filaments in the lower area of the clamping fixture, which frequently occur due to “specimen clamping”, could be significantly reduced with the aid of the described specimen preparation so that the majority of the specimens failed due to a largely homogeneous load application in the area of the clear length. In this context, it should be noted that the filaments are not in direct contact with the test plates, but are exclusively enclosed by the adhesive bed within the milled grooves (Figure 4b,c).

The pre-tensioning force only had to be reduced to 15 N in the case of long-term conditioning at a temperature of 50 °C, as the multifilament yarns embedded in the sodium hydroxide and potassium hydroxide solution sometimes failed before the defined pre-tensioning force of 150 N was reached. The tensile speed was increased to 6 mm/min once the pre-tensioning force had been reached. The test ended when a 90% drop in force was detected by the machine.

## 3. Results

Basically, it can be seen that all chemical aging processes follow the Arrhenius relationship. The effects on the samples are explained by way of example at lower or higher temperatures.

### 3.1. Nominal Cross-Sections

The gravimetrically determined nominal cross-sections *A_N_* of the unconditioned impregnated multifilament yarns SBR1_Reference_ and SBR2_Reference_ with a fiber cross-sectional area *A_F_* of approximately 0.24 mm^2^ were 0.97 ± 0.04 mm^2^ and 0.78 ± 0.03 mm^2^. The densities of the polymer structures including the air void content were 1.25 ± 0.02 g/m^3^ and 1.32 ± 0.02 g/cm^3^. The actual densities of the SBR1 and SBR2 impregnation systems will deviate from the values presented, as the density of the coating distorts the results. Nevertheless, the densities of the two impregnation systems can be (relatively) compared with each other.

The test results give the impression that the SBR1 impregnation system is initially more suitable for use in cementitious binders due to its higher nominal cross-section (higher protective effect). However, it should be noted that other material properties such as alkali and hydrolysis resistance or the heat resistance and density of the polymer structures play an important role in the assessment of the two impregnation systems (cf. Section 4).

### 3.2. Liquid Absorption Capacity

The impregnated multifilament yarns absorb liquid mainly through diffusive and capillary processes. At the same time, liquid penetrates intercurrently along defects (cracks or notches) into the filament cross-section (cf. Section 3.4). As a result of the liquid absorption, the material initially swells (bubble formation) and changes in volume. These physical processes are triggered by chemical conditioning and cause an increase in the mass of the samples. At the same time, the (alkaline) test liquids cause extraction of (water) soluble components from the polymer structure of the impregnation systems. Accordingly, the actual change in weight and volume is a balance of liquid absorption (weight increase) and dissolution processes (weight decrease). The intensity of the liquid absorption and the dissolution processes has a decisive effect on the protective effect of the SBR1 and SBR2 impregnation systems. Premature (high) liquid absorption of alkaline solutions into the polymer structures can promote an accelerated reduction in glass strength. The prerequisite for this is that these alkaline solutions penetrate through the impregnation cross-section to the outer film edges of the yarn. If additional extraction processes significantly perforate the polymer structures of the impregnation systems, alkaline solutions can accelerate the corrosion of the glass filaments and corrode them in the contact areas.

The samples CemFil-SBR1_NaOH, KOH_ stored in sodium hydroxide and potassium hydroxide solution already showed a significant weight increase of around 20 to 25 wt.-% on average after 24 h at a storage temperature of 23 °C. This weight increase remained almost unchanged until 192 h. This weight increase remained almost unchanged up to a period of 192 h, taking into account the error interval. Subsequently, between 672 and 4380 h, the samples showed a marginal increase in mass to approx. 30% by weight.

**Figure 5:**
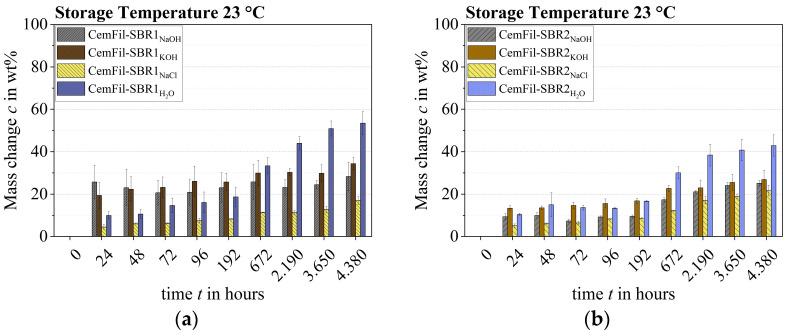
Change in mass of the impregnated multifilament yarns at 23 °C at the respective aging times: (**a**) CemFil-SBR1; (**b**) CemFil-SBR2.

In contrast, the increase in weight of the multifilament yarns stored in salt water and distilled water was lower at the beginning of chemical conditioning. The increase in weight after 24 h was between about 5 and 10 wt.-%. It is striking that the yarns stored in distilled water were about twice as heavy after 192 h as the samples conditioned in salt water. The water absorption capacity of the polymer structure SBR1 increased continuously over the storage period up to an average weight increase of 50% by weight after 4380 h. Consequently, the samples stored in distilled water had the highest weight increase. In contrast, the multifilament yarns stored in salt water generally had the lowest total weight increase (Figure 5a).

The CemFil-SBR2 multifilament yarns chemically conditioned at 23 °C essentially had a lower liquid absorption capacity. In particular, the samples stored in sodium hydroxide and potassium hydroxide solution showed a significantly lower weight increase over a period of 24 to 192 h compared to the yarns conditioned in salt water and distilled water. In the later course of the durability test, the masses of the differently stored samples did increase, but they did not reach the level of the CemFil-SBR1 samples. The conditioned multifilament yarns CemFil-SBR2_NaOH, KOH, and NaCl_ showed an increase in weight of between 20 and 30 wt.-% after 4380 h. The samples stored in distilled water had the highest weight increase with an average of just over 40 wt.-% (Figure 5b).

The CemFil-SBR1 impregnated yarns predominantly absorbed more liquid compared to the CemFil-SBR2 samples, which is partly due to the approximately 20% larger nominal cross-section of the impregnated filaments (cf. Section 3.1).

The presumed capacity of the impregnated multifilament yarns was reached between 2190 and 4380 h.

The ratios of the weight increases of the chemically conditioned multifilament yarns CemFil-SBR1 and CemFil-SBR2 were divergent at 50 °C. Basically, the samples stored in sodium hydroxide solution, potassium hydroxide solution, and saltwater reached their actual capacity after 24 h—with the exception of the samples stored in distilled water. These continued to increase in weight until they reach constant mass after about 192 h. It is noticeable that the CemFil-SBR2 multifilament yarns had an overall higher liquid absorption capacity. However, this can be explained by a more intensive degradation of the SBR1 impregnation system (cf. Section 3.3).

**Figure 6:**
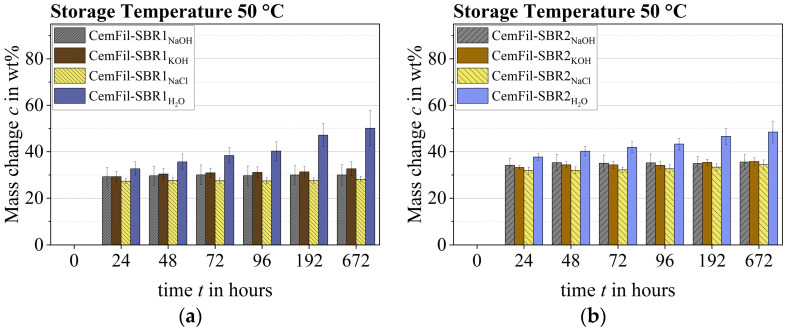
Change in mass of the impregnated multifilament yarns at 50 °C at the respective aging times: (**a**) CemFil-SBR1; (**b**) CemFil-SBR2.

All samples could no longer be weighed properly after 800 h at the latest, as the impregnation systems had begun to decompose (Figure 6). The decomposition processes led to the disintegration of filament groups of different sizes in the respective solutions.

At a storage temperature of 23 °C, the polymer structure SBR1 had a fundamentally higher liquid absorption capacity than the polymer structure SBR2 (cf. Figure 5). A storage temperature of 50 °C gave the impression that the chemically conditioned multifilament yarns CemFil-SBR1 had a lower liquid absorption capacity. However, this supposedly higher liquid absorption capacity of the CemFil-SBR2 samples was due to a more intensive degradation of the SBR1 polymer structure.

### 3.3. TGA

The corrosion processes in the glass structure triggered by the sodium hydroxide and potassium hydroxide solution caused mass losses, which were distorted by the mass reductions due to the extraction of water-soluble components from the polymer systems. 

The on-set temperatures of both polymer-impregnated multifilament yarns were within an almost identical temperature range, regardless of the conditioning scenarios. The melting process of the impregnation systems began in a temperature range from 383.04 ± 3.42 °C to 383.30 ± 3.15 °C. The decomposition of the polymer systems was completed in a temperature interval between 476.60 ± 4.46 °C and 476.32 ± 3.84 °C. The total mass loss *m_f_* of the yarns differed by about 8% (Table 2), which was again due to the different nominal cross-sections.

The changes in the mass of the impregnated multifilament yarns varied depending on the conditioning scenarios. After 28 days, the CemFil-SBR1 samples stored at 23 °C showed the greatest changes in mass. The yarns stored in sodium hydroxide solution, salt water, and distilled water lost almost 50% of their weight during this time. The exception is the samples exposed to potassium hydroxide solution, which showed a reduction in mass of around one-third. Subsequently, an increase in the weight of the yarns could be observed between six and twelve months, which again, with the exception of the samples stored in sodium hydroxide solution, almost reached the initial mass of the reference samples of around 48% by weight. At 51.99 ± 0.16% by weight, the multifilament yarns exposed to sodium hydroxide solution had the highest total mass loss after one year.

**Figure 7:**
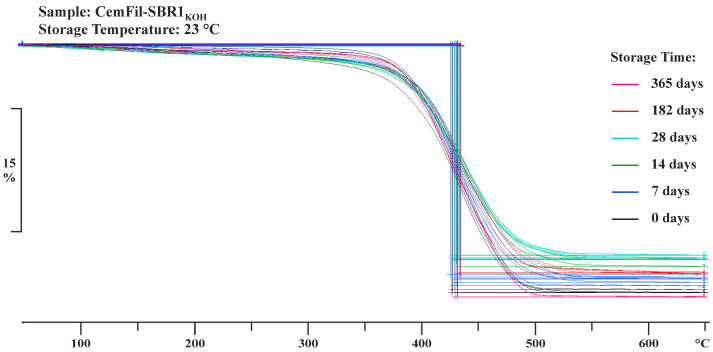
TGA measurement curves of the polymer-impregnated multifilament yarn CemFil-SBR1 stored at 23 °C in sodium hydroxide solution for up to one year. The mass changes were evaluated using the STARe Excellence software from Mettler Toledo (“step evaluation”).

The impregnated multifilament yarns CemFil-SBR2 had a maximum mass loss of approximately 11% by weight after 28 days at a storage temperature of 23 °C. In the following months, chemical conditioning led to an increase in mass, which was slightly higher than the total mass loss of the reference samples toward the end of storage. The CemFil-SBR2_NaOH_ samples had the highest mass change after one year at 43.36 ± 0.17% by weight (Figure 7).

**Figure 8:**
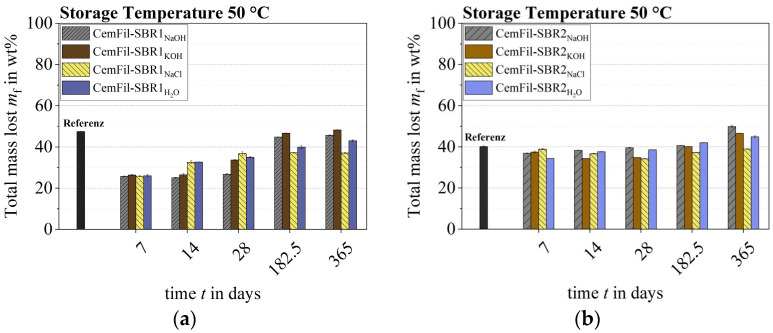
Loss of mass of the impregnated multifilament yarns at 50 °C at the respective aging times: (**a**) CemFil-SBR1; (**b**) CemFil-SBR2.

An increased storage temperature of 50 °C shortened the dissolution processes and liquid absorption by about 14 days. The soluble components of the polymer structure SBR1 were already extracted from the impregnation system after one week, which is why the liquid absorption dominated after 14 days until the end of chemical conditioning (Figure 8a). 

Depending on the different test liquids, the CemFil-SBR2 samples reached their highest mass reduction between 7 and 28 days (Figure 8b).

**Figure 9:**
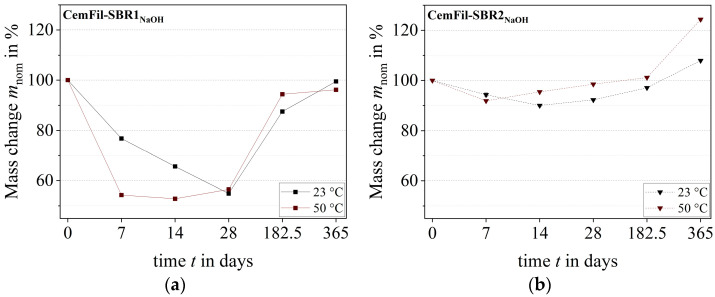
Standardized mass loss of the multifilament yarns stored in sodium hydroxide solution at 23 and 50 °C: (**a**) CemFil-SBR1; (**b**) CemFil-SBR2.

The liquid absorption capacity of the cavity volume (swelling capacity) in conjunction with diffusion and superimposed dissolution processes initially suggests that the polymer structure SBR2 had a higher capacity (cf. Section 3.2). The prerequisite for this would be that the degradation of the SBR2 polymer structure begins and is completed after just a few hours or days. Using the normalized mass loss of the differently impregnated multifilament yarns, it can be illustrated that the polymer structure SBR1 degraded more intensively and subsequently absorbed significantly more liquid than the impregnation system SBR2 (Figure 9). The direct comparison of the changes in mass between the reference and chemically conditioned samples also indicates this fact. 

The impregnated multifilament yarns CemFil-SBR1 had a higher mass loss due to a larger nominal cross-section, but this is not the only reason for the relative mass loss of around 50% by weight. The degradation of the polymer structure caused by the test liquids obviously played a much more important role (cf. Section 3.4).

It can generally be seen that the extent of the dissolution processes at the start of chemical conditioning was higher for the differently impregnated multifilament yarns than the swelling and diffusion capacity of the polymer structures. Toward the end of the storage process, liquid absorption predominated, which (after an initial reduction in mass) manifested itself in the form of an increase in mass.

The CemFil-SBR1 samples lost significantly more mass at the beginning of chemical conditioning than the CemFil-SBR2 yarns. These mass losses mean a more intensive degradation of the polymer structure, allowing alkaline media to reach the glass structure more easily. The increased liquid absorption capacity of the SBR1 polymer structure also accelerated possible glass corrosion. The impregnated multifilament yarns CemFil-SBR2 obviously had better alkali and hydrolysis resistance and a lower liquid absorption capacity in accordance with the changes in mass. The diffusion processes through the SBR2 impregnation cross-section were also slower than with the SBR1 polymer impregnation system due to the (minimally) higher material density (cf. Section 3.1).

### 3.4. Microscopy

The impregnated multifilament yarns CemFil-SBR1 mostly had a closed impregnation surface, which tended to blister slightly. Any accumulations of the polymeric-impregnating agent on individual filaments (agglomerations) were not recognizable.

Overall, the CemFil-SBR1 yarns were covered with an almost complete and even saturated impregnation film.

**Figure 10:**
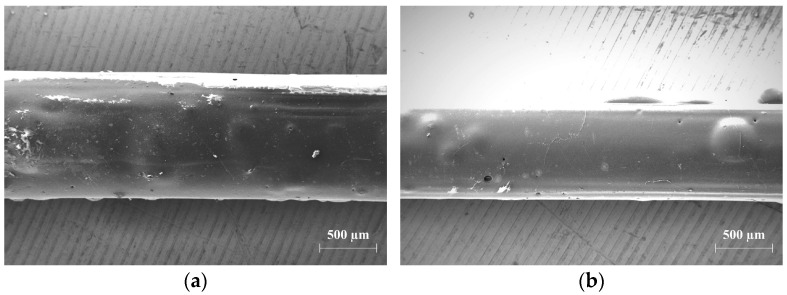
Details of an unconditioned multifilament yarn: (**a**) CemFil-SBR1: almost all individual filaments are coated with the SBR1 impregnation system; (**b**) CemFil-SBR2: the homogeneous polymer film tends to form bubbles.

The impregnated multifilament yarns CemFil-SBR2 were also wetted with a full-surface and homogeneous polymer film. However, the thinner polymer film tended to form more bubbles (Figure 10). The extent of blistering of the two impregnation systems decreased with increasing storage time until it was no longer observed after approximately one month.

Storage in sodium hydroxide and potassium hydroxide solution caused the undoubted degradation of the SBR1 impregnation system between 14 and 28 days at 23 °C, which was visible under the SEM. A recognizable degradation of the CemFil-SBR1 samples stored at 23 °C in salt and distilled water only began after 28 days. 

**Figure 11:**
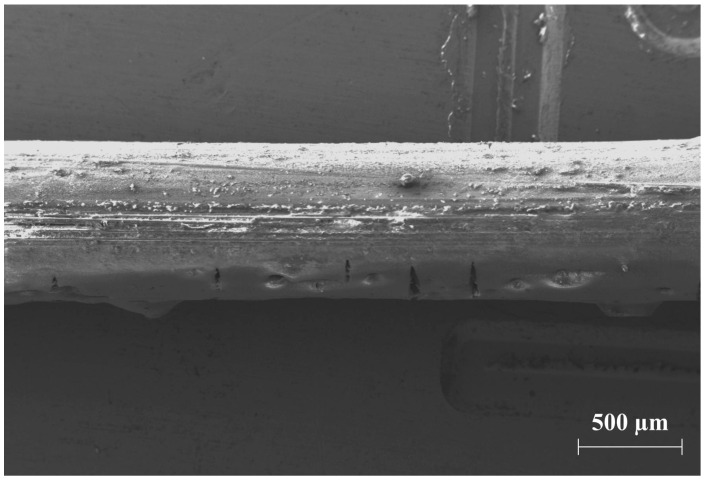
SEM image of a multifilament yarn of the type CemFil-SBR1, which has been exposed to potassium hydroxide solution at a temperature of 50 °C for a period of 28 days. The degradation of the polymer structure causes the exposure of individual filaments and the formation of cracks of different sizes.

The polymer system SBR1 was so damaged due to the extraction processes that a large number of filaments were no longer covered by a protective polymer film (Figure 11). The result of the exposed glass fibers was a reduction in the fiber–matrix adhesion, which is why the mechanical properties of the material deteriorated (cf. Section 3.5).

In addition, the aging processes accelerated by chemical conditioning caused a change in the physical-mechanical properties. The oxidation of the rubber phase of the polymeric impregnation system SBR1 led to post-crosslinking, which resulted in embrittlement or hardening of the polymer structure. As a result, the stiffened rubber phase was no longer able to absorb larger amounts of energy or form crazes. Furthermore, stresses arise between the impregnation surface and the multifilament core [37,38]. The result was the formation of cracks in the impregnation system, which became longer, wider, and deeper as the conditioning period continued. The crack lengths were between approximately 110 and 670 µm. In addition, swelling of the polymer structure occurred due to liquid absorption. The swelling caused residual stresses in the polymer surface, which also promoted the formation of cracks. The test liquids can penetrate through the cracks to the exposed filaments and damage them irreversibly.

The CemFil-SBR2 multifilament yarns embedded in the alkaline test liquids did not show any noticeable signs of dissolution, which would indicate a progressive degradation of the polymer system. However, chemical conditioning also triggered the formation of cracks. The crack lengths were limited to around 30 to 550 µm. Accordingly, the extent of crack formation was clearly less than in the CemFil-SBR1 samples. 

As a general rule, the longer the crack, the deeper and wider it is.

**Figure 12:**
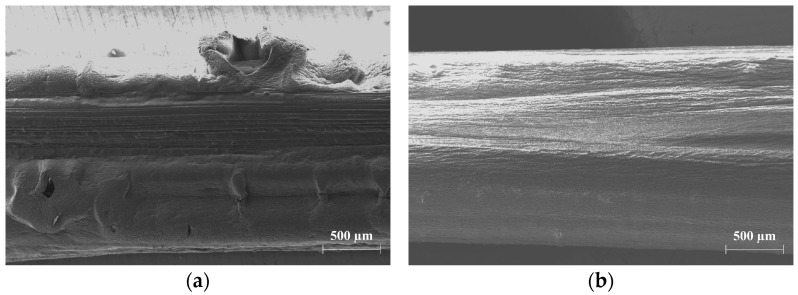
(**a**) The polymer structure of the SBR1 impregnation system is porous, wrinkled, and swollen after 28 days in distilled water at a temperature of 50 °C; (**b**) the morphology of the polymer structure of SBR1 is hardly changed after one year in 50 °C distilled water.

The storage of the impregnated multifilament yarns CemFil-SBR1 and CemFil-SBR2 in distilled water at 50 °C caused the formation of a porous, folded, and swollen polymer structure after just 28 days (Figure 12a). The appearance described was much more pronounced in the CemFil-SBR1 samples, which is why a large number of filaments were exposed. In contrast, the SBR2 impregnation system enclosed almost all of the yarns even after one year at a storage temperature of 50 °C (Figure 12b). The described characteristics were only observed in the multifilament yarns CemFil-SBR1 and CemFil-SBR2 stored in distilled water.

**Figure 13:**
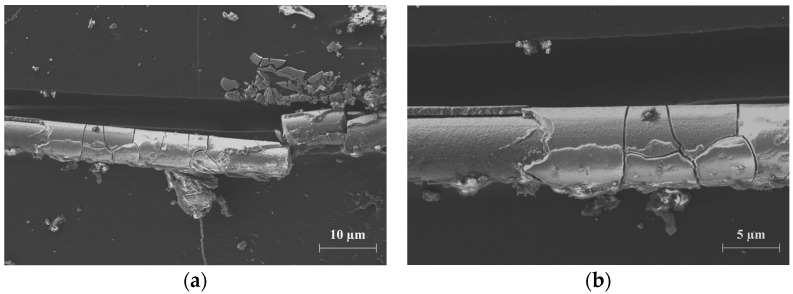
(**a**) Formation of shell-like clods around an exposed glass filament after 28 days in sodium hydroxide solution at 23 °C; (**b**) close-up of the characteristic corrosion phenomenon after 28 days in 2.5 percent sodium hydroxide solution at a temperature of 23 °C.

The chemical conditioning of the impregnated multifilament yarns in sodium hydroxide solution caused a loss of adhesion in the exposed yarns and apparently characteristic corrosion, which can be seen between 14 and 28 days depending on the temperature. The filaments in contact with the sodium hydroxide solution formed shell-like clods on the glass surfaces (Figure 13). This corrosion layer of almost the same thickness detached from the glass surface, which is why the filament cross-section was inevitably reduced.

According to the work of Scheffler et al., the corrosion layer has an increased zirconium content. Compared to the glass chemistry, the corrosion layer is also depleted in sodium; moreover, no potassium can be detected [39]. Furthermore, the silicon content in the corrosion product is significantly lower, which can be attributed to the degradation of the glass network.

It should be noted that Scheffler et al. chemically conditioned the alkali-resistant, non-impregnated multifilament yarns in 4 percent sodium hydroxide solution at 60 °C, and the corrosion phenomenon was observed after seven days [39]. Accordingly, a low-concentration (two percent) sodium hydroxide solution at lower temperatures of 50 °C also triggered this corrosion phenomenon after 14 days (albeit with a time delay).

### 3.5. Fiber Strand Tensile Tests

The non-chemically conditioned reference samples CemFil-SBR1 and CemFil-SBR2 had tensile strengths of 1241 ± 65 and 1046 ± 46 N/mm^2^, respectively. As the individual filaments of the tested yarns were not parallel to each other (ondulation), the partially corrugated filaments were loaded differently during the tensile tests. This resulted in irregular, successive, and sporadic failure of individual filaments along the entire free test length in the cross-section of the test specimens. Therefore, the determined strengths of the impregnated multifilament yarns varied considerably in some cases, which is why the error was correspondingly large [40].

The higher tensile strength values of CemFil-SBR1 compared to CemFil-SBR2 can be explained by neglecting the chemical compositions of the two impregnation systems with the aid of the different nominal cross-sections. The non-impregnated multifilament yarns had tensile strengths of 82 ± 54 N/mm^2^. 

**Figure 14:**
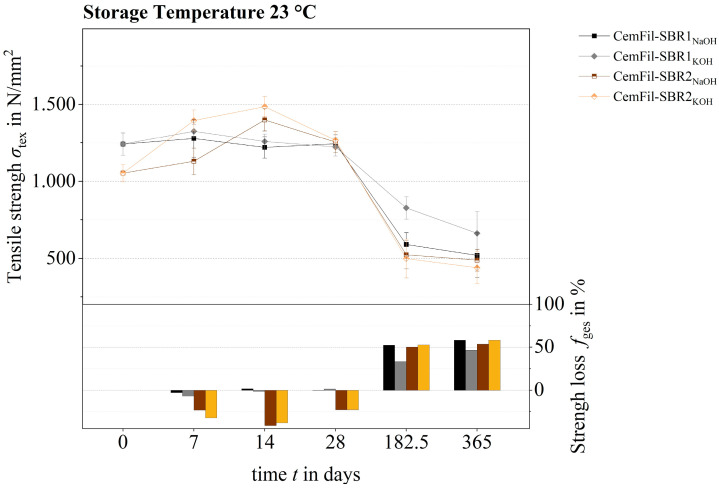
Tensile strength development and loss of strength of samples CemFil-SBR1 and -SBR2 chemically conditioned in sodium hydroxide and potassium hydroxide solution over a period of 365 days at a storage temperature of 23 °C.

Taking into account the partly widely scattered strength values of the different tested samples, the impregnated multifilament yarns CemFil-SBR1 had similar tensile strengths to their reference samples after 28 days at a conditioning temperature of 23 °C. Accordingly, the degree of strength loss also showed no significant changes (Figure 14). Similarly, the tensile strengths of the CemFil-SBR1 samples stored in salt and distilled water were unchanged.

**Figure 15:**
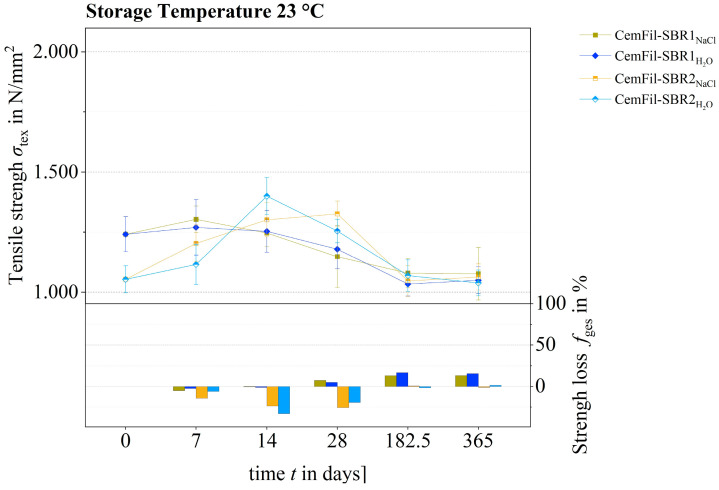
Tensile strength development and loss of strength of samples CemFil-SBR1 and -SBR2 chemically conditioned in salt and distilled water over a period of 365 days at a storage temperature of 23 °C.

The CemFil-SBR2 multifilament yarns conditioned under the same conditions clearly had higher tensile strength values after 7 and 14 days compared to their reference samples (Figure 15). 

The increase in strength of the CemFil-SBR2 samples stored in sodium hydroxide solution was slower than that of the multifilament yarns conditioned in potassium hydroxide solution. The increase in strength of the CemFil-SBR2 samples exposed to salt and distilled water was almost identical and did not change noticeably after 28 days. An incipient reduction in the tensile strength of the CemFil-SBR2_NaOH, KOH_ samples can be observed for the first time after 28 days. This reduction in strength is due to the incipient corrosion of the filaments.

Nevertheless, the tested tensile strengths of these samples were still significantly increased, even when the measurement error interval is taken into account. The maximum tensile strengths of the CemFil-SBR2 multifilament yarns were reached after 14 days.

The thermal-oxidative degradation of the rubber phase can cause the formation of further cross-links, which increase the cross-linking density. For this reason, various mechanical properties of the material can temporarily improve at the start of thermal oxidation [41]. The cross-linking density of styrene-butadiene rubbers in particular is known to increase in the initial phase of (artificial) weathering and decrease again later on [42].

Over a period of between 28 days and 1 year, the tensile strength of the CemFil-SBR1 and CemFil-SBR2 samples stored in sodium hydroxide and potassium hydroxide solution gradually decreased. The percentage loss of strength after one year was between 45 and 60%.

Taking into account the error interval, the tensile strengths of the CemFil-SBR2 multifilament yarns impregnated with salt and distilled water were largely unchanged. In contrast, the yarns impregnated with the SBR1 impregnation system lost strength, which is due to the degradation of the polymer structure (cf. Section 3.3).

The chemical effect of water can lead to the cleavage of hydrolyzable groups from the main chain of the impregnation system. This cleavage causes molecular weight degradation, which results in a deterioration of the mechanical properties of the polymer. Normally, the degree of damage caused by water in combination with catalytically active bases (or acids) is greater than the damage to the polymer structure caused by the chemical effect of water alone. In this case, no clear differentiation could be made between the degree of damage caused by salt and distilled water due to the discrepancy in the measurements and the resulting approximately identical loss of strength.

The increased storage temperature of 50 °C accelerated the loss of strength of the impregnated multifilament yarns as well as the chemical aging processes of the polymer structures (post-crosslinking, extraction) by the test liquids.

The degree of strength loss of the CemFil-SBR1 and Cemfil-SBR2 samples stored in sodium hydroxide and potassium hydroxide solution was already over 50% after 28 days. All samples were almost completely corroded after one year, which is why the percentage loss of strength was almost 100%. Accordingly, the chemically conditioned yarns could not be tested properly. The majority of the multifilament yarns stored for up to one year failed before reaching the pre-tensioning force of 15 N, so no meaningful strength values could be measured.

The CemFil-SBR1 samples conditioned in salt and distilled water had strength losses of between 14 and 17%, which can be attributed to the degradation of the impregnation systems. The percentage loss of strength of the CemFil-SBR2 yarns stored in the same media was between 3 and 6%.

The reduced tensile strength due to the degradation of the polymer structures correlates with the mass loss of the SBR1 impregnation system. A correlation between the loss of strength and mass was not recognizable in the CemFil-SRB2 samples due to the post-crosslinking.

## 4. Conclusions

The SBR1 and SBR2 impregnation films applied to the multifilament yarns using an impregnation system wet almost all of the individual filaments, which is why they were completely enclosed by a polymer protective layer. This result is not a matter of course, as multifilament yarns impregnated with styrene-butadiene rubbers often form agglomerations [43]. The formation of agglomerations means that a large number of filaments are not wetted with the impregnating agent. This, in turn, can at best increase the displacement stiffness of a textile, but neither increase the tensile strength nor improve the durability.

The nominal cross-section of the chemically unconditioned reference samples CemFil-SBR1 with 0.97 ± 0.04 mm^2^ was larger compared to the reference samples CemFil-SBR2 with 0.78 ± 0.03 mm^2^, which theoretically allows a higher capacity as well as a greater weight loss. Depending on the chemical conditioning scenarios, the SBR1 impregnation system actually had a higher liquid absorption capacity, although it appears that the weight increase in the CemFil-SBR2 samples was greater due to diffusive and capillary processes. This illusion is created by the more intensive degradation and the associated higher weight reduction of the polymer structure SBR1. The percentage loss in mass of the embedded, impregnated multifilament yarns CemFil-SBR1 compared to the reference samples was a maximum of around 21.62% by weight. The CemFil-SBR2 samples, on the other hand, lost a maximum of 5.75% by weight. 

According to these results, the CemFil-SBR2 samples have better alkali and hydrolysis resistance and a better protective effect. 

The tensile strengths of the chemically unconditioned reference samples CemFil-SBR1 were higher than those of the reference samples CemFil-SBR2 due to the larger nominal cross-section.

The majority of the tested tensile strengths of epoxy resin-impregnated multifilament yarns by Büttner were in a strength range between 1120 ± 71 N/mm^2^ and 1370 ± 74 N/mm^2^ [43]. The highest tensile strength achieved in his work was 1700 ± 96 N/mm^2^ for an impregnation polymer based on epoxy resin. In addition, Büttner examined multifilament yarns impregnated with a styrene-butadiene rubber [43]. However, in this case, no increase in load-bearing capacity could be determined during the uniaxial tensile tests. At 780 ± 52 N/mm^2^, the tensile strength of these samples was within the strength range of non-impregnated reference samples. Scanning electron microscopic examinations showed that the impregnation at most “glues” individual filaments together. Accordingly, only a few filaments were involved in the load transfer, which explains the lower tensile strength values. Furthermore, the durability cannot be improved due to the poor impregnation quality. The yarns tested by Büttner were AR glass direct rovings of type LTR 5325 and a fineness of 2400 tex from Owens Corning in Toledo (Ohio), United States of America [43]. The clear length of the tested multifilament yarns was 150 mm; the test speed was set at 7.5 mm/min.

In the work of Scheffler, AR glass fibers of the type VET ARG from Vetrotex (Saint-Gobain Group) in Paris, France, with a fineness of 640 tex were tested, which were impregnated with carboxylated, thermoplastic styrene-butadiene [12]. The impregnation led to an increase in strength of approximately 35%. The free stretching length of the multifilament yarns was 500 mm. The strength of the samples was tested at 200 mm/min. 

The non-impregnated reference samples CemFil-SBR1_Reference_ and CemFil-SBR2_Reference_ had tensile strengths of 1241 ± 65 N/mm^2^ and 046 ± 46 N/mm^2^, respectively. These measured values are approximately in the same strength range as the epoxy resin-impregnated multifilament yarns investigated by Büttner [43]. The non-impregnated multifilament yarns had tensile strengths of 828.56 ± 53.56 N/mm^2^. Accordingly, the application of the two impregnation systems SBR1 and SBR2 resulted in an increase in the tensile strength of the multifilament yarns of approximately 21% and 33%, respectively, compared to the non-impregnated yarns.

Depending on the chemical conditioning scenarios, the polymer-impregnated CemFil-SBR1 samples had the highest liquid absorption capacity. This result converges with the test results from Kempis [33]. However, the CemFil-SBR1 specimens had a larger nominal cross-section, which generally leads to a higher load-bearing capacity increase.

In conclusion, the high impregnation quality inevitably led to an improvement in the durability and an increase in the tensile strength of the impregnated multifilament yarns CemFil-SBR1 and CemFil-SBR2. However, the results of the durability tests varied significantly in some cases.

A textile impregnated with the SBR2 impregnation system is probably more suitable for use in cementitious binders. However, the results presented in this paper are insufficient for an accurate assessment of the performance of the two differently impregnated composite materials in concretes.

It is conceivable that the CemFil-SBR1 samples or a textile impregnated with the SBR1 polymer system may be more suitable due to the larger nominal cross-section. Regardless of the lower alkali and hydrolysis resistance, the larger nominal cross-section of the SBR1 polymer system in principle causes a delay in the diffusion and capillary processes of the alkaline concrete pore solution. In addition, a stronger impregnation additionally protects the filaments from damage caused by crystallization processes during hydration [19,20].

The effect of these two factors should be investigated in more detail with regard to the technical service life of polymer-impregnated textiles in cementitious binders. Further durability tests are therefore planned to examine this issue with textiles impregnated with the aqueous polymer dispersions SBR1 and SBR2.

## Figures and Tables

**Table 1:** List of the test liquids used in the investigations.

Test Liquid	pH Value
2.5 percent sodium hydroxide solution (NaOH_aq_)	13.4
2.5 percent potassium hydroxide solution (KOH_aq_)	13.6
3.0 percent natrium chloride solution (NaCl_aq_)	9.1
distilled water (H_2_O_dest._)	7.0

**Table 2:** Total mass loss of the impregnated multifilament yarns, CemFil-SBR1 and CemFil-SBR2.

Impregnated Multifilament Yarn	Total Mass Loss mf in [wt.-%]
CemFil-SBR1	47.79 ± 0.16
CemFil-SBR2	39.96 ± 0.47

## Data Availability

Data are contained within the article.
